# Enhancing cardiac postoperative care: a smartwatch-integrated remote telemonitoring platform for health screening with ECG analysis

**DOI:** 10.3389/fcvm.2024.1443998

**Published:** 2024-09-23

**Authors:** Rosangela Monteiro, Guilherme C. M. Rabello, Camila R. Moreno, Matheus S. Moitinho, Fábio A. Pires, Nelson Samesina, Luiz Antônio M. César, Flávio Tarasoutchi, Fábio Fernandes, Pietro C. C. O. Martins, Bruna M. Mariano, Alexandre de M. Soeiro, Adriana Palhares, Carlos Alberto Pastore, Fabio B. Jatene

**Affiliations:** ^1^ Department of Cardiovascular Surgery—InovaInCor, Instituto do Coracao, Hospital das Clinicas HCFMUSP, Faculdade de Medicina, Universidade de São Paulo, São Paulo, Brazil; ^2^ Biomedical Informatics Laboratory, Instituto do Coracao, Hospital das Clinicas HCFMUSP, Faculdade de Medicina, Universidade de São Paulo, São Paulo, Brazil; ^3^ Electrocardiography Unit, Instituto do Coracao, Hospital das Clinicas HCFMUSP, Faculdade de Medicina, Universidade de São Paulo, São Paulo, Brazil; ^4^ Chronic Coronary Disease Unit, Instituto do Coracao, Hospital das Clinicas HCFMUSP, Faculdade de Medicina, Universidade de São Paulo, São Paulo, Brazil; ^5^ Valvular Heart Disease Clinical Unit, Instituto do Coracao, Hospital das Clinicas HCFMUSP, Faculdade de Medicina, Universidade de São Paulo, São Paulo, Brazil; ^6^ Cardiomyopathy-Aortic Diseases Clinical Unit, Instituto do Coracao, Hospital das Clinicas HCFMUSP, Faculdade de Medicina, Universidade de São Paulo, São Paulo, Brazil; ^7^ InCor Emergency Unit, Instituto do Coracao, Hospital das Clinicas HCFMUSP, Faculdade de Medicina, Universidade de São Paulo, São Paulo, Brazil

**Keywords:** wearables, smartwatch, heart surgery, telemonitoring, electrocardiography, atrial fibrillation

## Abstract

**Aims:**

The integration of smartwatches into postoperative cardiac care transforms patient monitoring, systematically tracking vital signs and delivering real-time data to a centralized platform. This study focuses on developing a platform for seamless integration, assessing reliability, and evaluating the impact on post-cardiac surgery. The goal is to establish a robust foundation for understanding the efficacy and dependability of smartwatch-based telemonitoring, enhancing care for this population.

**Methods and results:**

A total of 108 cardiac surgery patients were divided into telemonitoring (TLM) and control (CTL) groups. The TLM group utilized smartwatches for continuous monitoring of vital parameters (SpO_2_, HR, BP, ECG) over 30 ± 3 days. Statistical analyses (Pearson, Intraclass Correlation, Bland-Altman, Tost Test) were employed to compare smartwatch measurements with traditional methods. Significant correlations and concordance were observed, particularly in HR and BP measurements. Challenges were noted in SpO_2_ measurement. The ECG algorithm exhibited substantial agreement with cardiologists (Kappa: 0.794; *p* > 0.001), highlighting its reliability. The telemonitoring platform played a crucial role in early detection of clinical changes, including prompt Emergency Department (ED) visits, contributing significantly to preventing outcomes that could lead to mortality, such as asymptomatic Atrioventricular block. Positive patient responses affirmed technological efficacy, especially in identifying cardiac arrhythmias like atrial fibrillation.

**Conclusion:**

The integration of smartwatches into remote telemonitoring for postoperative cardiac care demonstrates substantial potential, improving monitoring and early complication detection, thereby enhancing patient outcomes. The FAPO-X Study (Assisted Digital Telemonitoring with Wearables in Patients After Cardiovascular Surgery; NCT05966857) underscores the promising role of telemonitoring in postoperative cardiac care.

## Graphical Abstract

Enhancing Cardiac Postoperative Care: A Smartwatch-Integrated Remote Telemonitoring Platform for Health Screening and ECG Analysis. **(1)** Smartwatch ECG: Samsung Galaxy 5 captures ECG in post-cardiac surgery patients over 30 days for continuous monitoring and preliminary evaluation by a cardiologist. **(2)** Patient Journey: **(A)** Preoperative consultation; **(B)** Cardiac surgery; **(C)** Postoperative assessment with Smartwatch data acquisition; **(D)** Conventional care for comparison; **(E)** Remote Telemonitoring integrates daily health data into the Hospital's EHR; **(F)** Clinical team analyzes data for teleconsultations and interventions; **(G)** Return to the institution based on clinical evolution, if necessary.

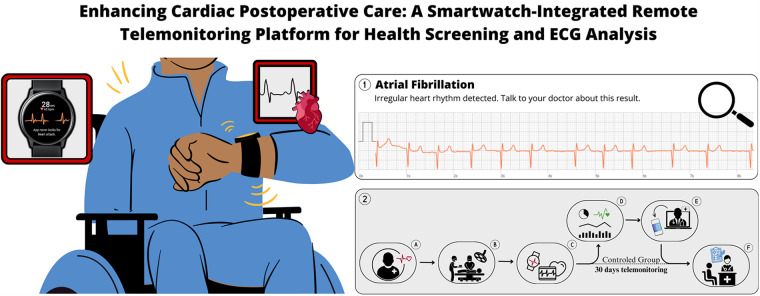

## 1 Introduction

In the contemporary landscape of healthcare, technological advancements have significantly transformed patient care, particularly in monitoring and surveillance. Wearable devices equipped with multifunctional sensors have emerged as crucial tools for comprehensive health data acquisition. This convergence of technology and healthcare holds profound implications, especially for individuals recovering from cardiac surgery, who face various postoperative challenges, including hemodynamic instability, infectious complications, and cardiac arrhythmias (1, 2). Simultaneously, telemonitoring remote technologies represent a promising avenue for extending postoperative care beyond traditional healthcare settings, although their substantive application in advanced healthcare research remains limited (3).

A critical consideration in this context is the temporal dynamics of postoperative atrial fibrillation, with a significant proportion of episodes occurring within the initial days following surgery, coinciding with heightened systemic inflammation induced by the surgical insult (4). Consequently, there is a compelling need for continuous monitoring throughout the convalescent phase, necessitating innovative strategies to bridge the gap between hospital-based care and home recovery. Wearable technologies, such as smartphones and smartwatches, hold potential as indispensable tools for real-time surveillance and seamless care transitions for postoperative cardiac patients.

In light of these considerations, this study aims to investigate the feasibility and benefits of integrating wearable technology into postoperative care protocols for cardiac surgery patients, alongside assessing the precision and reliability of data collected. Additionally, it seeks to compare patient journeys with and without smartwatch integration, aiming to refine postoperative care paradigms and enhance patient outcomes following cardiac surgery.

## 2 Methods

### 2.1 Study population

Conducted at InCor, University of São Paulo, from May 2022 to June 2023, this interventional study received approval from the Ethics Committee for Research Project Analysis (CAPPesq), under the protocols SDC 5.874.032 and CAAE 66520122.0.0000.0068. The study enrolled 108 post-operative cardiac surgery patients during their pre-surgical consultation. To ensure an accurate assessment of the digital platform's efficacy among users, patients lacking digital usability or declining to use the smartwatch were assigned to the control group. Other patients were randomly allocated in a 1:1 ratio using block randomization to ensure equal sample sizes across the telemonitored and control groups over time, enhancing comparability and reducing bias, thus improving study validity. The sample composition of the different study groups and eligibility criteria are described below:

• **Telemonitored (TLM, *n* = 55):** Patients who received telemonitoring with a SAMSUNG™ Galaxy Watch5 Smartwatch and Web FAPO-SI^3^ platform, which is integrated with the hospital's electronic medical record system).
• **Control (CTL, *n* = 53):** Patients who underwent standard treatment at the institution without any wearable monitoring.

The study included individuals aged 22 and above with surgical indications for heart conditions. Inclusion criteria covered mitral or aortic valve diseases, coronary artery disease, or aortic disease. Participants needed to be willing to adhere to procedures and provide written consent. Exclusion criteria applied to those without cardiac surgery, post-surgical complications leading to hospitalization exceeding 14 days, interference with smartwatch data acquisition, wrist skin conditions, and adverse reactions to device materials. The study, registered under Clinical Trials code NCT05966857, complied with Brazil's General Data Protection Regulation (LGPD no. 13.709/2018).

### 2.2 Study design

This single-center interventional study comprised six phases. Phase 1 involved preoperative consultations, including comprehensive anamnesis and study information. In Phase 2, eligible patients assigned the Informed Consent Form (ICF), prepared for surgery, and had their demographic data collected. Phase 3 focused on collecting intraoperative data after cardiac surgery.

Phase 4 consisted of a postoperative assessment upon patient transfer to the ward from the ICU, where health measures (blood pressure (BP), heart rate (HR), electrocardiogram (ECG), and peripheral oxygen saturation (SpO_2_) were recorded using both the smartwatch and reference devices.

Following discharge, Phase 5 implemented a 30 ± 3-day telemonitoring period using the smartwatch, recording health data three times daily and triggering medical interventions through the FAPO-SI^3^ platform.

In Phase 6, patients returned for outpatient consultations at InCor, where health measures were again collected using the smartwatch and reference devices. A technology perception questionnaire was administered, and ECG classifications by the smartwatch were verified by a specialized cardiologist.

The Control Group (*n* = 53), comprising patients with heart disease who underwent standard procedures, had data described as pre-follow-up (Phase 4) and post-follow-up (Phase 6). The Telemonitoring Group (TLM) had data described as pre-telemonitoring and post-telemonitoring.

**Figure 1:**
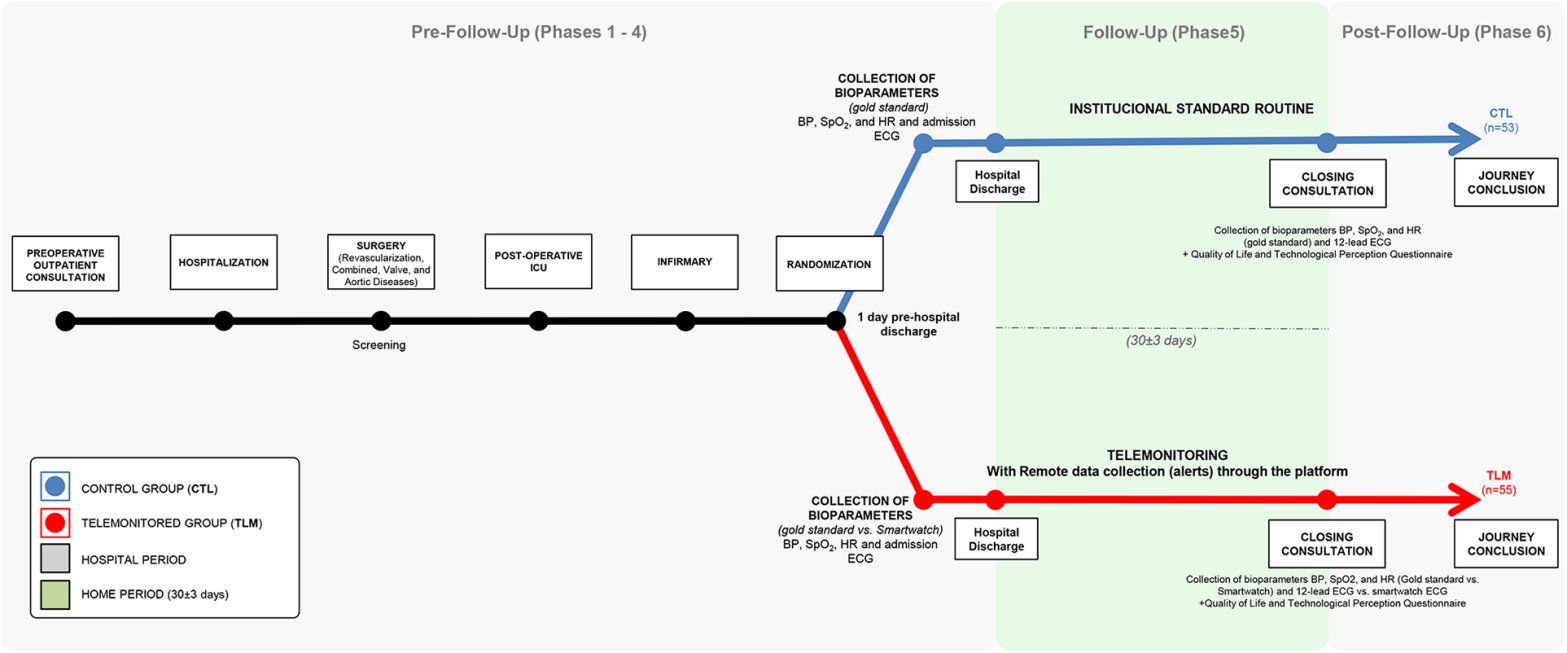
Illustration of study design, timeline and methods.

The timeline of the Patient Journey for both groups is presented in Figure 1.

### 2.3 Development of the Web FAPO-Si^3^ telemonitoring platform

The Web FAPO-SI^3^ telemonitoring platform was designed for collecting vital health data from patients via smartwatches. Using an Android API developed for the study, data from the Samsung Health® and Health Monitor® applications were extracted in real-time and connected to a PostgreSQL database in an AWS EC2 instance. Extracted data were loaded into the InCor Institutional Electronic Health Record (EHR) twice daily using a Json extractor. The Web FAPO-SI^3^ platform within the EHR displayed telemonitored patients' data in order of criticality, with red indicating the most critical, followed by yellow.

**Figure 2:**
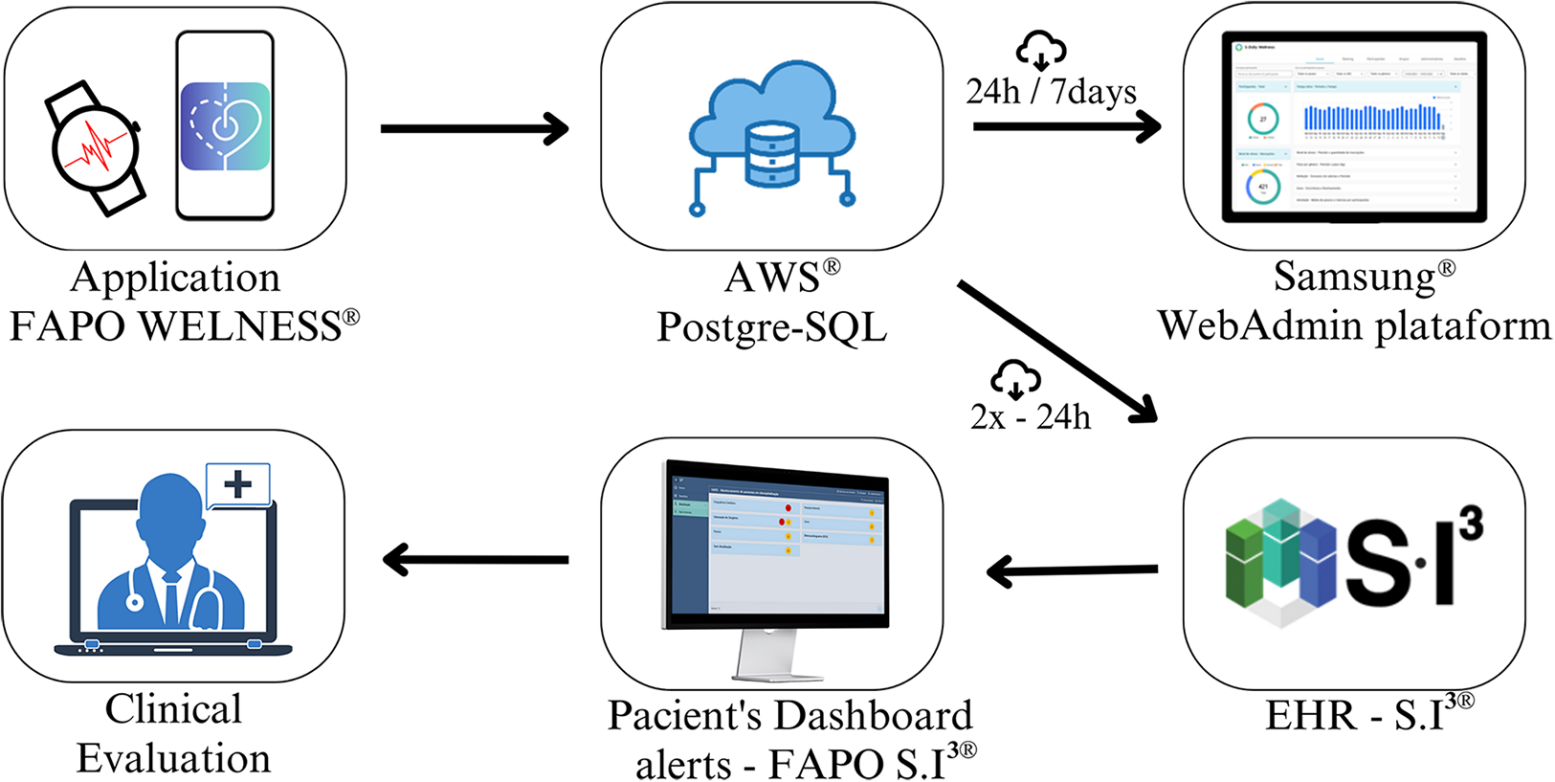
Description of the capture, storage and management flowchart raw health data in the study. Amazon Web Services (AWS) and HER SI^3^ (InCor Institutional Electronic Health Record).

For patient follow-up in Phase 5, the WhatsApp Application® facilitated communication and teleconsultations. Healthcare professionals accessed acquired data twice a day, allowing continuous monitoring and early detection of abnormal situations based on pre-defined criteria triggering notifications and alerts of varying criticality levels (Table 1). The data flow process is illustrated in Figure 2.

**Table 1:** Clinical alerts received through the Web FAPO-SI^3^ telemonitoring platform.

Parameter	Color alert	Criteria
Blood Pressure	Normal Parameters	Systolic [100–140 mmHg] and diastolic [70–90 mmHg]
	Yellow Alert	Systolic [100–180 mmHg] and diastolic [90–110 mmHg]
	Red Alert	Systolic [<90 and >180 mmHg] and diastolic [<60 mmHg and >100 mmHg].
Heart Rate	Normal parameters	HR detection between 60 and 100 bpm.
	Red Alert	Detection of HR < 50 bpm outside sleep period; or HR > 100 bpm (concurrently assessed by watch)
Oxygen Saturation	Normal parameters	95%–100%
	Yellow Alert	91%–95%
	Red Alert	<90%

### 2.4 Standard ECG vs. smartwatch ECG

During phases 4 and 6 of the study, participants underwent resting ECG exams concurrently with the collection of ECG data from a smartwatch. This screening aimed to assess the cardiac rhythm of the patients. The resting ECGs were conducted at a speed of 25 mm/s with a calibration of 1 mV/10 mm using an outpatient MAC 2000 ECG Machine (GE Medical Systems Information Technologies, Inc., WI, USA). Electrodes were positioned in the classic 12-lead configuration and a clinical cardiologist evaluated these parameters, following the electrocardiographic report guidelines set by the Brazilian Society of Cardiology, to exclude arrhythmias (5). Participants were instructed to perform ECG measurements three times daily using the smartwatch, or when requested.

### 2.5 Standard HR measurement vs. smartwatch HR

In Phases 4 and 6, smartwatch HR measurements followed the manufacturer's guidelines. Three consecutive resting HR measurements were then alternately performed using both the smartwatch and the standard method. The smartwatch utilized photoplethysmography (PPG), emitting light through the skin to monitor reflected light variations, correlated with heartbeats^5^. An internal algorithm calculated real-time HR from detected systolic peaks. In contrast, the gold standard HR measurement employed the OMRON model HEM-7122 digital BP monitors by measuring blood BP.

### 2.6 Standard BP measurement vs. smartwatch BP

In Phase 4, the smartwatch was calibrated for BP measurement as per the manufacturer's instructions. Subsequently, 8 resting BP measurements were taken, alternating between the smartwatch and the standard OMRON model HEM-7122 device. Standard device measurements included supine readings after 10 min of rest (3 consecutive readings), one seated measurement, and one orthostatic measurement, with a 30 s interval between the latter two. Phase 6 replicated this methodology. Additionally, participants performed smartwatch blood pressure measurements three times daily or as prompted by the onboard app.

### 2.7 Standard SpO_2_ vs. smartwatch oximetry

In Phases 4 and 6, SpO_2_ values were measured concurrently using the smartwatch and a digital pulse oximetry device (Fingertip-Bewine®). The conventional oximetry method employed infrared light to assess heart rate and peripheral oxygen levels. Participants performed smartwatch SpO_2_ measurements three times daily or as directed by the app. If unsuccessful initially, up to 10 additional attempts were made. Participants unable to obtain readings after 10 attempts were advised to wear the smartwatch overnight for subsequent follow-up assessments.

### 2.8 Statistical modelling

The study utilized REDCap® for data storage and administered questionnaires for clinical and sociodemographic details. Clinical parameters were measured conventionally and with a smartwatch at enrollment and 30-day follow-up, with duplicate measurements. A technological impact assessment questionnaire was included.

Data analysis involved presenting characteristics and ECG reports using proportions and descriptive statistics. Various statistical tests (*t*-test, Mann–Whitney, Chi-square, Fisher's Exact test) analyzed differences and associations. Multiple Correspondence Analysis visualized data patterns.

Linear correlations and method reliability were assessed using Pearson Correlation and Intraclass Correlation Coefficient (ICC). Two-way ANOVA examined time and device effects on health parameters. Concordance was evaluated through One-Sample t-test and the Bland-Altman method. Equivalence between devices was tested with an independent samples *t*-test using the Two One-Sided Tests (TOST) approach. Missing data were imputed assuming Missing Completely at Random (MCAR). JAMOVI® Version 2.3 and SPSS® Version 21 were used for analysis, with significance set at *p* < 0.05.

## 3 Results

### 3.1 Baseline characteristics

**Figure 3:**
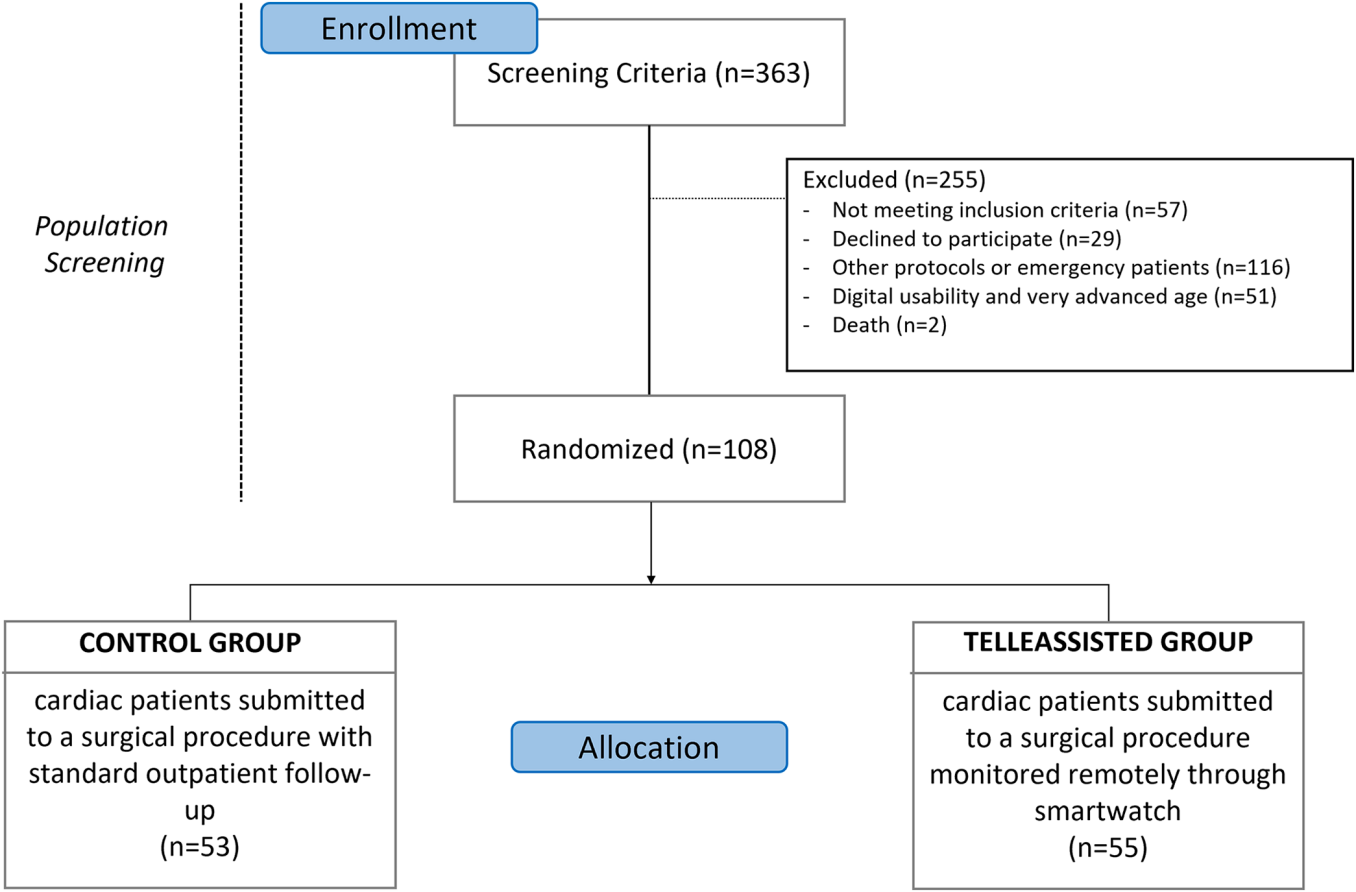
Study protocol flow diagram.

The study reviewed participants' medical records to assess cardiac surgery history, study outcomes, and exclusions. Out of 363 eligible patients, 108 were selected after screening (Figure 3). Among 182 approached, 71 (39.01%) were excluded; notably, 38 (53.52%) were from the control, and 33 (46.48%) from the telemonitored group. Gender-wise, 34 (47.89%) were female, and 52 (73.24%) identified as white.

Surgical patient analysis (CTL = 53, TLM = 55) revealed a significant age difference (*t*: 6.55, *p* < 0.001). Despite this, the TLM group showed a diverse age range, including elderly patients but with a higher concentration of younger participants (33–76 years).

Comparisons between groups, using tests, showed no significant differences in gender, ethnicity, surgery type, duration, comorbidities, and education, confirming comparability (Table 2).

**Table 2:** Baseline characteristics of the 108 patients studied in different groups.

Patients Characteristics	CTL (*n* 53)	TLM (*n* 55)	*p-*value
Age (years) mean ± stand. deviation	64.22 ± 8.29	52.71 ± 9.92	*<0*.001*
Male gender (*n*), %	(30) 56.6%	(33) 60.0%	*0*.*720*^#^
Skin Color (*n*), %			*0*.*825*^##^
White	(43) 81.1%	(41) 74.5%	
Black	(3) 5.7%	(4) 7.3%	
Brown	(7) 13.2%	(9) 16.4%	
Yellow	(0) 0.0%	(1) 1.8%	
Type of surgery (*n*), %			*0*.*087*^##^
Revascularization	(24) 45.3%	(18) 32.7%	
Valve	(28) 52.8%	(29) 52.7%	
Aorta's Diseases	(1) 1.9%	(6) 10.9%	
Combined	(0) 0%	(2) 3.6%	
Surgery Time (hours) mean ± stand. deviation	04:43 ± 1:08	04:56 ± 1:19	*0*.415*
Comorbidities (*n*), %			
Altered Heart Rhythm	(30) 56.6%	(36) 65.5%	*0*.*284*^#^
Prior Myocardial Infarction	(10) 18.9%	(5) 9.1%	*0*.*121*^#^
Heart Failure	(15) 28.3	(19) 34.5%	*0*.*572*^#^
Cerebral Vascular Disease	(2) 3.8%	(2) 3.6%	*1*.*000*^###^
Hypertension	(33) 62.3%	(32) 58.2%	*0*.*554*^#^
Dyslipidemia	(29) 54.7%	(24) 43.6%	*0*.*172*^#^
Diabetes Mellitus	(8) 14.5	(7) 12.7	*0.862* ^###^
Alcoholism	(20) 37.7%	(28) 50.9%	*0*.*166*^#^
Smoking	(15) 28.3%	(14) 25.5%	*0*.*782*^#^
BMI median (interquartile range)	26.6 (19.4–41.8)	27.7 (17.4–39.3)	*0*.620*
Education degree (*n*), %			*0*.*134*^##^
Incomplete Primary Education	(19) 35.8%	(17) 30.9%	
Complete Primary Education	(6) 11.3%	(4) 7.3%	
Incomplete High School	(3) 5.7%	(5) 9.1%	
Complete High School	(10) 18.9%	(10) 18.2%	
Incomplete Higher Education	(3) 5.7%	(1) 1.8%	
Complete Higher Education	(8) 15.1%	(18) 32.7%	
Information not available	(4) 7.5%	(0) 0%	

Total, CTL: control, and TLM:telemonitored. Results are given as mean (SD) or number (%) of subjects. All values of the characteristics, except age are expressed in number of patients (*n*) and percentage.

* *T*-test.

** *U*-Mann–Whitney.

^#^ Chi-square.

^##^ Fisher's Exact.

^###^ Chi square with Yate's correction.

### 3.2 Patient's allert: frequency of clinical alerts in the FAPO-Si^3^ application for the TLM group

**Figure 4:**
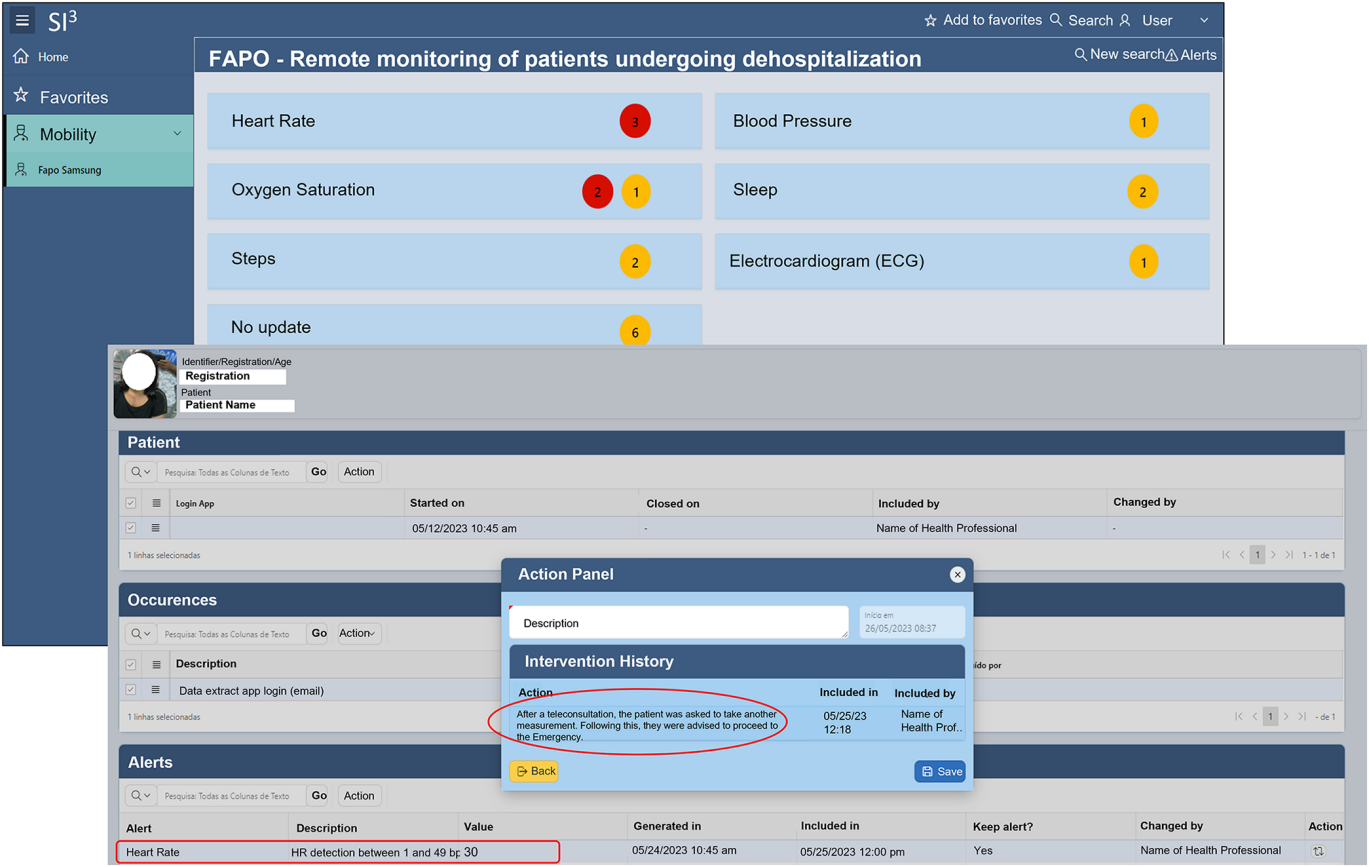
FAPO-SI^3^ professional health monitoring interface: screenshots display parameter classification with active alerts and severity levels on the initial screen. The second screen provides dynamic visualization of patient health parameters and integrated alert history from the electronic health record, supporting interventions and personalized teleconsultations.

A customized dashboard was integrated into the EHR SI^3^ using raw patient data and the FAPO-SI^3^ Wellness app, categorizing health metrics by criticality (red for the highest, yellow for lower) (Figure 4). This facilitated real-time monitoring and trend identification. In Phase 5, patients used WhatsApp daily for communication and teleconsultations, sharing clinical status, symptoms, and ECG reports from Samsung Health via smartwatch. Throughout Phase 5, the FAPO-SI^3^ platform generated 17,572 alerts, with heart rate anomalies (56.4%) and SpO_2_ alerts (18.7%) being prominent. Red alerts accounted for 63.9% of events.

### 3.3 Analysis of emergency department visits in patients with remote telemonitoring vs. institutional standard routine

The analysis of the number of patients visits to the Emergency Department (ED) was conducted comparing the two study groups. In the CTL group, 35 patients did not visit the ED and 12 patients did. In the TLM group, 36 patients did not visit the ED and 12 patients did.

The *χ*^2^ test with Continuity Correction was used to evaluate the association between the both groups in the ED visits and indicated no statistically significant association between group (CTL or TLM) and the ED visits [*χ*^2^_(1)_ < 0.001; *p* = 1.000] (Supplementary Table S2).

**Figure 5:**
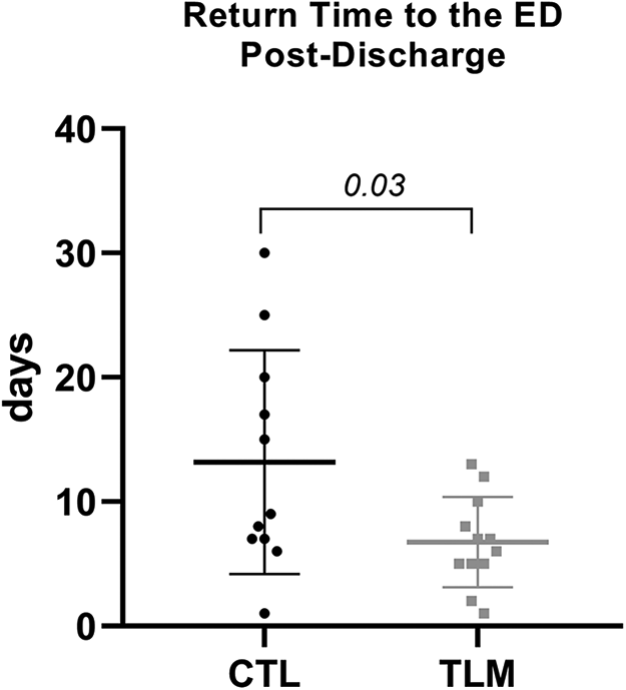
Comparison of mean return times to the emergency department between telemonitored patients and control group: graph demonstrating the average return times to the emergency department between telemonitored patients (TLM, *n* = 12) and the control group (CTL, *n* = 12). Error bars represent the standard deviation around the mean. Statistical analysis using a *t*-test indicates a significant difference (*p* = 0.032) between the groups.

Furthermore, the analysis of the mean return time to the emergency department revealed a statistically significant difference between TLM and those in the CTL (*t*-test, *p* = 0.032). Patients in the TLM group exhibited a mean return time of 6.75 ± 3.62 days, whereas patients in the CTL group demonstrated a mean return time of 13.18 ± 8.98 days (Figure 5).

### 3.4 Atrial fibrillation detection: evaluating smartwatch vs. medical reports for heart rhythm concordance

In our cohort with 53 participants, 30.2% manifested atrial fibrillation (AF). Two participants were excluded due to pacemaker-related data inconsistencies. The concordance analysis between telemonitored ECGs and medical reports demonstrated a compelling Kappa coefficient of 0.794 (*p* < 0.001) for AF and sinus rhythm detection (Table 3). This analysis highlighted that out of 714 ECGs (17.7%) identified as AF and 3,034 ECGs (75.4%) as sinus rhythm, the overall agreement between medical and smartwatch reports reached 93.1%. This proportion signifies the proportion of correct classifications relative to the total sample size. Importantly, the smartwatch demonstrated a notably high case detection rates for both conditions, underlining its reliable identification proficiency. Furthermore, the predictive metrics displayed outstanding a notable accuracy (93.1%), specificity (92.4%), and sensitivity (96.2%) in AF detection (Tables 4, 5 and Figure 5). These comprehensive findings underscore the effectiveness and reliability of the statistical analyses, emphasizing the affirmative impact and potential of smartwatch technology in the assessment of heart rhythm.

**Table 3:** Assessing concordance: Kappa test analysis of smartwatch vs. clinical physician interpretation for ECGs in TLM group telemonitoring period (Phase 5).

Smartwatch vs. medical report					
			Medical report (*n* = 4,026)		Total
			Sinus rhythm	Atrial fibrillation	
Smarwatch (*n* = 4,026)	Sinus Rhythm	Count	3,034	28	3,062
		Expected Count	2497.7	564.3	3062.0
		% Total	75.4%	0.7%	76.1%
	Atrial Fibrillation	Count	250	714	964
		Expected Count	786.3	177.7	964.0
		% Total	6.2%	17.7%	23.9%
	Total	Count	3,284	742	4,026
		Expected Count	3284.0	742.0	4026.0
		% Total	81.6%	18.4%	100.0%
Agreement Measure Kappa		**Value**	**Asymp. std. error.**	**T aprox.**	**Sig. Aprox.**
		***0***.***794***	0.012	51.086	***>0***.***001***

The cells display category counts, with totals for each category and an overall count. The Kappa value (*n* 4,026) gauges agreement between Smartwatch and Medical Report, accompanied by statistical measures, including asymptotic standard error, T approximation, and significance level.

The bold values specifically indicate the *p*-values that demonstrate statistical significance in the comparison.

Italicized values indicate the Kappa coefficient (0.794) and the associated statistical significance (*p* < 0.001).

**Table 4:** Contingency table for smartwatch vs. clinical physician interpretation for ECGs in TLM Group Telemonitoring Period.

Recoded data for decision test statistics	Gold positive	Gold negative	Total
AF patients (*n* = 16)			
Smart positive test	714	30	744
Smart negative test	28	575	603
Total	742	605	1,347
Without AF patients (*n* = 37)^a^			
Smart positive test^a^	2,459	141	2,600
Smart negative test^a^	220	28	248
Total	2,679	169	2,848
Total (*n* = 53)			
Smart positive test	714	250	964
Smart negative test	28	3,034	3,062
Total	742	3,284	4,026

^a^ For The analysis of patients without AF, the focus was on the diagnosis of sinus rhythm, given the absence of an AF report for the specialist physician.

**Table 5:** Confusion matrix for smartwatch vs. clinical physician interpretation for ECGs in TLM group telemonitoring period.

Matrix	AF patients (*n* = 16)	Without AF patients (*n* = 37)*	Total (*n* = 53)
Sensitivity^a^	96.23%	91.79%*	96.23%
Specificity^b^	95.04%	16.57%*	92.39%
Accuracy^c^	95.69%	87.32%*	93.09%
Prevalence^d^	55.09%	94.07%*	18.43%
Positive Predictive Value^e^	95.97%	94.58%*	74.07%
Negative Predictive Value^f^	95.36%	11.29%*	99.09%
Post-test Disease Probability^g^	95.97%	94.58%*	74.07%
Post-test Health Probability^h^	95.36%	11.29%*	99.09%
Positive Likelihood Ratio	19.41	1100.00*	12.64
Negative Likelihood Ratio	0.0397	0.4957*	0.04085

*Notably, for patients without AF, a confusion matrix was employed, with emphasis on diagnosing sinus rhythm due to the absence of an AF report from the specialist physician.

^a^ Sensitivity.

^b^ Specificity.

^c^ Accuracy

^d^ Disease prevalence

^e^ Positive predictive value

^f^ Negative predictive value

^g^ Post-test probability of having disease, and

^h^ Post-test probability of being healthy. Notably, for patients without AF, a confusion matrix was employed, with emphasis on diagnosing sinus rhythm due to the absence of an AF report from the specialist physician.

### 3.5 Effect of technological intervention on health measures: comparative analysis of CTL and TLM groups

In the TLM group, a Two-Way ANOVA assessing “time” and “devices” interaction for Systolic Blood Pressure (S-BP), (Dyastolic Blood Pressure) D-BP, HR, and SpO_2_ revealed significant differences. Notably, D-BP showed a significant difference (*p* = 0.007) between Phase 4 (76.5 ± 9.5) and Phase 6 (80.6 ± 11.2). HR exhibited a highly significant difference (*p* < 0.0001) between Phase 4 (90.6 ± 11.8) and Phase 6 (82.4 ± 14.6). Similarly, SpO_2_ demonstrated a highly significant variance (*p* < 0.0001) between Phase 4 (91.2 ± 2.8) and Phase 6 (93.8 ± 3.6).

In the CTL group with gold standard acquisitions, a paired *t*-test indicated no statistically significant S-BP differences between Phase 4 (113.1 ± 16.51) and Phase 6 (120 ± 19.18) (*p* = 0.058). However, D-BP exhibited a significant difference (*p* = 0.01), with mean values of (71.39 ± 9.95) in Phase 4 and (76.29 ± 10.25) in Phase 6. Similarly, HR showed a significant difference (*p* = 0.006) between Phase 4 (84.96 ± 14.39) and Phase 6 (78.94 ± 18.47). SpO_2_ displayed a significant difference (*p* = 0.003) between Phase 4 (93.24 ± 2.98) and Phase 6 (95.43 ± 3.16).

### 3.6 Comparing smartwatch data to gold standard: pearson correlation coefficient and ICC TLM group health data

Correlations between standard and smartwatch methods were examined during Phase 4 and 6. Phase 4 exhibited very strong positive correlations for S-BP, D-BP, and HR (*r* = 0.979, *p*: <0.001; *r* = 0.963, *p*: <0.001; *r* = 0.976, *p*: <0.001, respectively). In Phase 6, a strong positive correlation was observed for HR (*r* = 0.840, *p*: <0.001), and S-BP, D-BP, and HR showed strong positive correlations (*r* = 0.781, *p*: <0.001; *r* = 0.623, *p*: <0.001; *r* = 0.840, *p*: <0.001, respectively). No significant correlations were found for SpO_2_ in both phases (*r* = −0.027, *p* = 0.847; *r* = 0.006, *p* = 0.963).

In the TLM group, ICC analysis indicated excellent agreement for S-BP, D-BP, and HR in both Phase 4 (ICC: 0.990, *α*: 0.989, *p* < 0.001; ICC: 0.981, *α*: 0.981, *p* < 0.001; ICC: 0.987, *α*: 0.988, *p* < 0.001, respectively) and Phase 6 (ICC: 0.877, *α*: 0.877, *p* < 0.001; ICC: 0.770, *α*: 0.767, *p* < 0.001; ICC: 0.909, *α*: 0.909, *p* < 0.001, respectively). SpO_2_ measurements did not show significant ICC values in both Phase 4 (ICC: −0.037, *α*: −0.054, *p*: 0.577) and Phase 6 (ICC: 0.008, *α*: 0.011, *p*: 0.484).

Grouping all standard and smartwatch measurements across phases revealed excellent agreement (ICC: 0.981, *α*: 0.981, *p* < 0.001).

### 3.7 Comparing smartwatch data vs. the gold standard: TOST test and Bland–Altman plot analysis of TLM Group's health data

**Figure 6:**
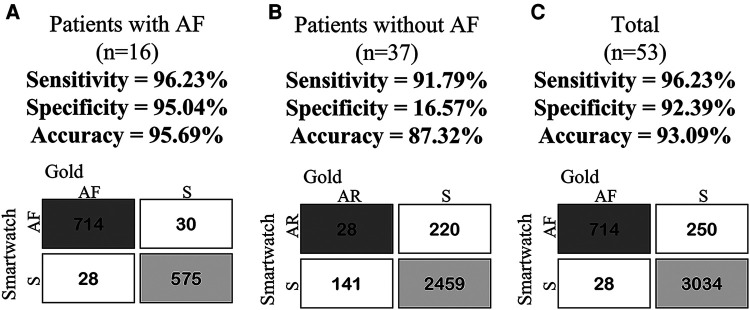
Confusion matrix comparing smartwatch vs. Clinical physician: AF, atrial fibrillation; AR, arrhythmias; S, sinus rhythm. **(A)** (Patients with AF), **(B)** (Patients without AF), and **(C)** (Total patients). In group **(B)**, focus is on diagnosing sinus rhythm and other rhythms due to the absence of an AF report from the specialist physician.

After addressing missing data with MCAR imputation, a Bland–Altman analysis assessed the reliability and comparability of S-BP, D-BP, HR, and SpO_2_ between the Phase 4 and the Phase 6. Single Sample *T*-tests during both phases showed that, for most variables, there were no significant differences between measurements from different devices, with *p*-values of 0.05, indicating overall measurement reliability (Table 6 and Figure 6).

**Table 6:** Bland–Altman analysis results from telemonitored group: smartwatch vs. gold standard.

Phase 4				
	S-BP (mmHg)	D-BP (mmHg)	HR (bpm)	SpO_2_ (%)
	*n* = 55	*n* = 55	*n* = 55	*n* = 55
Gold Standard (mean ± SD)	115 ± 13.8	76.5 ± 9.36	89.1 ± 12.3	94.0 ± 3.04
Smartwatch (mean ± SD)	116 ± 13.0	76.7 ± 9.00	90.6 ± 11.8	91.2 ± 2.82
Difference (mean ± SD)	0.700 ± 5.38	0.128 ± 4.19	1.53 ± 5.19	−2.75 ± 4.27
Mean (Between Devices)	115	76.6	89.8	92.6
Lower limit of agreement	−11.256	−8.354	−11.781	−5.63
Upper limit of agreement	9.856	8.098	8.65	11.12
Single Sample *T*-test (*p*-value)	0.3	0.8	**0**.**03**	**<0**.**001**
Phase 6				
	S-BP	D-BP	HR	SpO_2_
	*n* = 55	*n* = 55	*n* = 55	*n* = 55
Gold Standard (mean ± SD)	119 ± 14.3	80.6 ± 11.2	82.3 ± 16.6	96.3 ± 2.50
Smartwatch (mean ± SD)	118 ± 13.6	80.0 ± 11.2	82.4 ± 14.6	93.8 ± 3.59
Difference (mean ± SD)	−0.751 ± 9.3	−0.670 ± 9.6	0.115 ± 9.2	−2.45 ± 4.18
Mean (Between Devices)	119	80.3	82.4	95.0
Lower limit of agreement	−17.588	−18.310	−18.242	−5.70
Upper limit of agreement	19.089	15.15	18.011	10.60
Single Sample *T*-test (*p*-value)	0.4	0.6	0.9	**<0**.**001**

Bland–Altman analysis for S-BP, D-BP, HR, and SpO_2_ with *n* = 55 per variable. SD denotes standard deviation. Values are expressed as Mean ± SD.

The bold values specifically indicate the *p*-values that demonstrate statistical significance in the comparison.

Therefore, both phases exhibited robust measurements for most variables. Although discrepancies emerged in HR and SpO_2_ measurements during Phase 4, the original raw data indicated agreement in HR measurements. Agreement across all measurements of S-BP, D-BP, and HR was observed during both measurement periods, except for SpO_2_. Detailed data can be found in Supplementary Material (Table S2 and Figures S1, S2).

**Figure 7:**
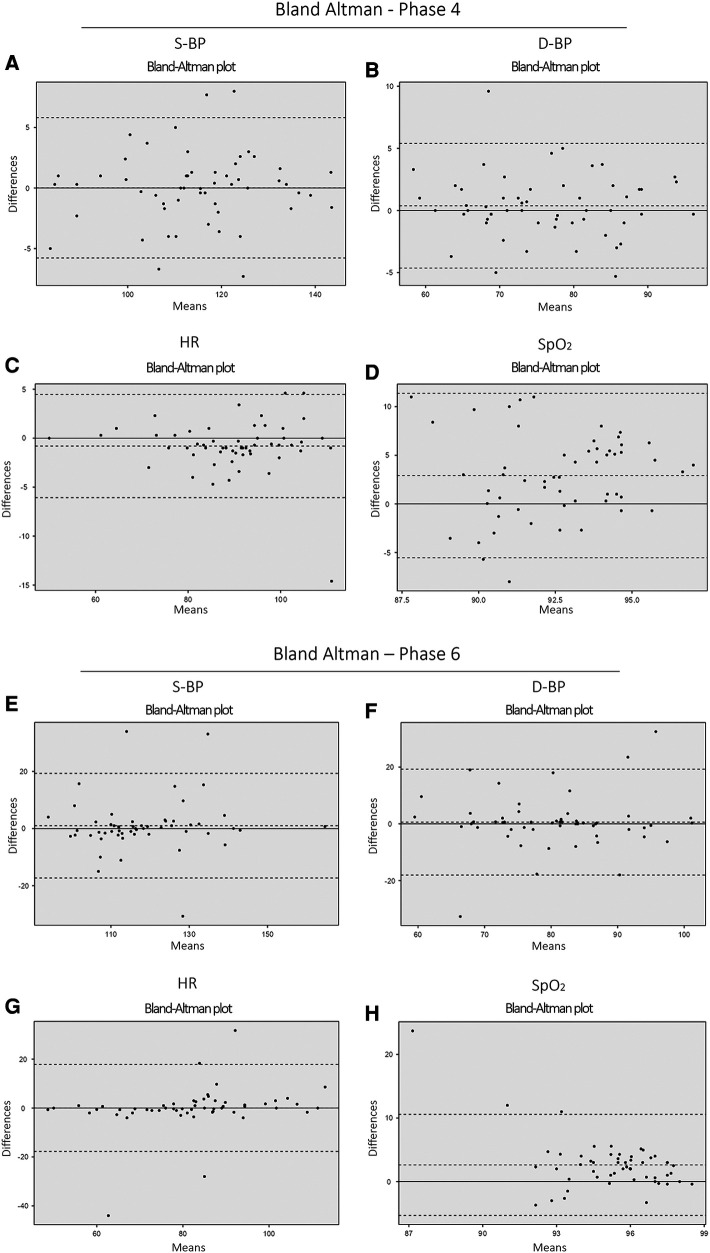
Bland–Altman analysis results for the telemonitored group (pre-telemonitoring and post-telemonitoring phases): smartwatch vs. Gold Standard Comparison. The plot showcases the agreement between measurements obtained from the smartwatch and the gold standard for various vital signs: [Phase 4 (pre-telemonitoring): **(A)** S-BP, **(B)** D-BP, **(C)** HR, and **(D)** SpO_2_] and [Phase 6: **(E)** S-BP, **(F)** D-BP, **(G)** HR, and **(H)** SpO_2_] (*n* = 55, per variable).

**Figure 8:**
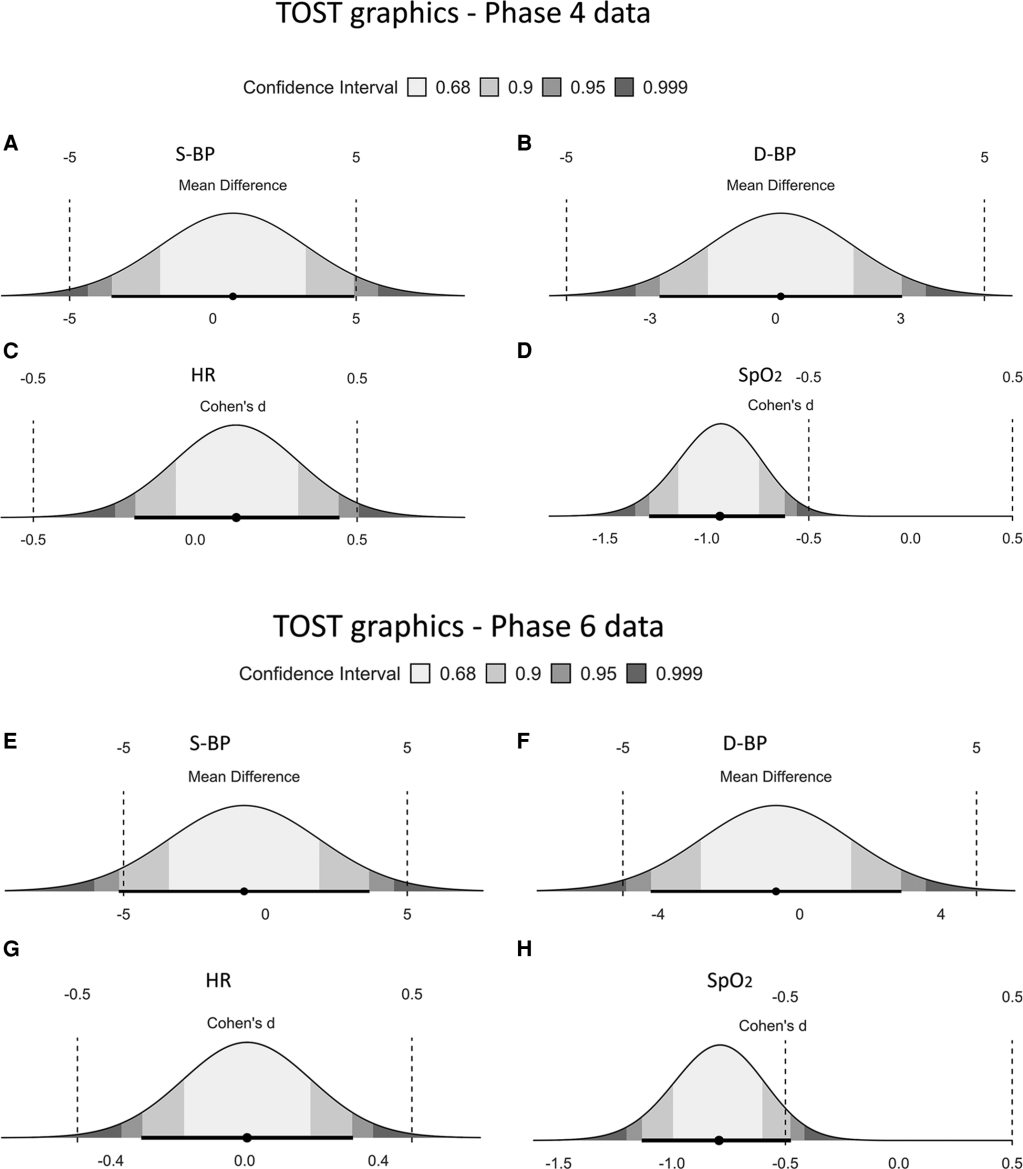
TOST graphs for Pre- and post-measurement phases: [phase 4: **(A)** S-BP, **(B)** HR, **(C)** D-BP and **(D)** SpO_2_] and [phase 6: **(E)** S-BP, **(F)** D-BP, **(G)** HR, and **(H)** SpO_2_]. Each subplot showcases the statistical and equivalence analysis for the vital signs.

The analysis of the equivalence of health measures showed that there were no statistically significant differences between the measures of S-BP, D-BP, and HR when comparing the traditional and smartwatch methods. However, there was a significant difference between the methods for SpO_2_ both for phase 4 and phase 6 [*p* < 0.001; *p*: <0.001], respectively. Furthermore, for the measures of phase 4 S-BP [*p* upper = 0.014, *p* lower = 0.048], D-BP [*p* upper = 0.02, *p* lower = 0.003], and HR [*p* upper < 0.001, *p* lower = 0.027] was significant equivalence of the methods. In the measures of phase 6 D-BP [*p* upper: 0.023; *p* lower: 0.005] and HR [*p* upper: 0.004; *p* lower: 0.006], indicated equivalence between the methods, but, for the S-BP measure, it is noteworthy that the lower *p*-value [*p* lower: 0.048] was found to be significant, although the upper *p*-value [*p* upper: 0.057] was not (Table 7 and Figure 7). However, it is important to highlight that when analyzing the original raw data, equivalence was observed for S-BP in phase 6, as well as for all measures of S-BP, D-BP, and HR for both measurement periods (Figure 8). Detailed data can be accessed in Supplementary Tables S4, S5 and Figures S3, S4.

**Table 7:** TOST results for telemonitored in the phase 4 and phase 6—vital signs during telemonitored.

TOST results phase 4		*t*	df	*p*
S-BP	*t*-test	0.274	108	*0*.*785*
	TOST Upper	2.23	108	***0***.***014***
	TOST Lower	−1.68	108	***0***.***048***
D-BP	*t*-test	0.0731	108	*0*.*942*
	TOST Upper	2.93	108	***0***.***002***
	TOST Lower	−2.78	108	***0***.***003***
HR	*t*-test	0.666	108	*0*.*507*
	TOST Upper	3.29	108	***<***.***001***
	TOST Lower	−1.96	108	***0***.***027***
SpO_2_	*t*-test	−4.91	108	***<0***.001*
TOST results phase 6		*t*	df	*p*
S-BP	*t*-test	−0.282	108	0.779
	TOST Upper	1.60	108	0.057
	TOST Lower	−2.16	108	**0**.**017**
D-BP	*t*-test	−0.314	108	0.754
	TOST Upper	2.03	108	**0**.**023**
	TOST Lower	−2.65	108	**0**.**005**
HR	*t*-test	0.0387	108	0.969
	TOST Upper	2.66	108	**0**.**004**
	TOST Lower	−2.58	108	**0**.**006**
SpO_2_	*t*-test	−4.16	108	**<0**.001*

* Interpretation of TOST Upper and Lower only when *t*-test is not significant.

The bold values specifically indicate the *p*-values that demonstrate statistical significance in the comparison.

Italicized values represent the p-values, indicating the statistical significance of the comparisons.

### 3.8 Technology perception variations and self-care implications: insights from group analysis

The collected data from the technology perception questionnaire reveals significant differences and similarities among groups. In terms of technology usage, “Computer and mobile phone” is the most common choice for all patients (50.9%), followed by “Television and radio” (37.7%), and “Do not use technology” (9.4%). Self-care perception varies, with the CTL group (65.5%) highlighting “Gave me more control”, in contrast to the TLM group (32.1%).

Perceived benefits of the FAPO study also differ notably. The response “More information” is more prevalent in the CTL group (73.6%) than the TLM group (34.5%). Regarding the FAPO-X watch and app features, there's equivalence: 70.9% in the CTL group and 27.3% in the TLM group find them “very useful”. Messages from the ChatBot via Whatsapp® are seen as useful by both groups (58.2% CTL, 32% TLM, marked “Very useful”).

**Figure 9:**
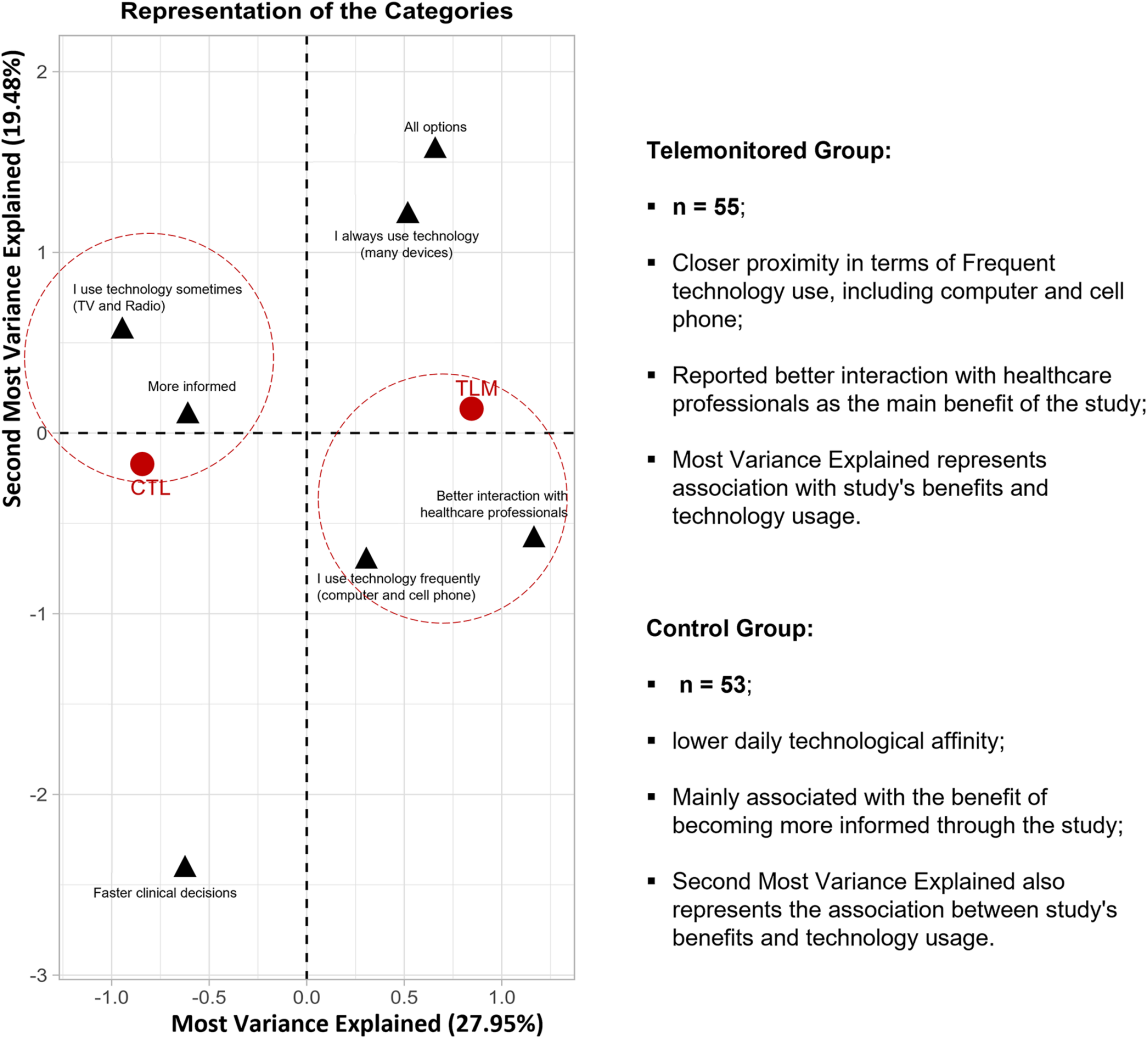
B-Plot: perception of the technological impact: Correspondence analysis illustrates relationships between variables, showing positive correlations in clustered groups and negative correlations on opposite sides of the origin. Variable distance from the origin indicates significance on the map, with greater distance representing more prominence.

These findings highlight diverse technology use patterns, recognized self-care, and FAPO study benefits. Through MCA, two main dimensions account for 47.4% of variable distance variations. The TLM group is closely linked to frequent tech use, especially computers and phones, suggesting greater technological interaction. They report better healthcare professional interaction. The CTL group is less tech-oriented and associates more with study-related information gain. Biplot axes reflect these dimensions (*R*^2^: 0.61, *p* < 0.001; *R*^2^: 0.34, *p* < 0.001) for benefits and tech use. Similarly, “Second most variance explained” (*R*^2^: 0.73, *p* < 0.001; *R*^2^: 0.52, *p* < 0.001) also represents these aspects (Figure 9).

## 4 Discussion

The study investigates the viability of integrating smartwatches into a telemonitoring platform for patients in post-cardiac surgery recovery. It is grounded in the growing importance of home monitoring, particularly for cardiovascular parameters such as BP, HR, and cardiac arrhythmias, which have gained significant attention in the telemonitoring domain (6).

This technology is regarded as crucial for lowering medical costs, facilitating the expansion of clinical care beyond hospital confines (7). Through our analysis of ED visits, we observed comparable frequencies between the CTL and TLM groups, indicating no statistically significant difference. While telemonitoring does not directly reduce ED visits, its benefits extend beyond immediate metrics. Data analysis highlights telemonitoring's capability for early detection of clinical changes, enhancing symptom recognition and management through continuous surveillance. This proactive approach facilitates timely interventions, potentially preventing exacerbations and optimizing patient outcomes. Furthermore, patients benefit from enhanced education on symptom recognition and management, potentially reducing unnecessary healthcare utilization. This integrated approach is increasingly integrated into telemedicine and healthcare institutions, supporting extended home monitoring for complex cardiovascular patients (8–11).

Among health data extracted from innovative health devices, ECG data stands out due to its fast and convenient acquisition by these devices. The literature underscores the telemonitoring benefits linked to devices capable of early detection of cardiac arrhythmias like AF and atrial tachycardias (12, 13). Focusing on ECG readings through smartwatch integration, demonstrated high concordance and accuracy in detecting AF, emphasizing the device's reliability in clinical applications.

This capability is crucial as conventional monitoring practices may overlook transient or asymptomatic arrhythmias, which can lead to delayed interventions. Hospital protocols often involve 48 to 72 h of continuous monitoring postoperatively, especially for patients with clinical indications or procedures carrying intermediate to high risk of AF (14). However, this approach may not capture the full scope of postoperative cardiac electrical activity over extended periods, resulting in gaps in documentation and medical records, and potentially affecting patient outcomes within 30 days (15).

Our study groups were well-matched in terms of gender, ethnicity, type of surgery, duration of surgery, and comorbidities, ensuring comparability. This matching is advantageous as it highlights the higher incidence of AF observed in the telemonitored group, which exceeds the postoperative AF rates typically reported in the literature. Studies have shown that postoperative AF rates are generally around 14% in cardiac surgery patients (16). In our study, however, the telemonitored group exhibited a higher AF incidence of 17%. This increased detection is likely due to the enhanced capabilities of the smartwatch, even with intermittent ECG monitoring. If continuous ECG monitoring were available, it is plausible to infer that the incidence rate could be even higher. Therefore, there is a pressing need for technological advancements by device manufacturers to support continuous monitoring, which could further improve the detection and management of postoperative arrhythmias.

The implementation of this technology depends on the participant's home internet access, which remains a significant barrier in many regions, including Brazil (17, 18). To address this issue, our institution provided mobile internet access to research participants, demonstrating improvements in monitoring and health outcomes. The literature underscores the need for global improvements in internet access to make such approaches universally applicable (19, 20).

There is substantial evidence that telemonitoring can improve health outcomes, leading to early detection of complications, better treatment adherence, and improved clinical outcomes (21, 22). However, it is crucial to have backup plans ready for device failure or erroneous readings, such as verification protocols, data redundancy, and efficient technical support.

The cost of implementing and maintaining these technologies is an important aspect. Although initially high, studies suggest that telemonitoring can be economically viable in the long term for both public and private healthcare systems, due to the reduction in hospitalizations and severe complications (23, 24). Cost-benefit analyses are necessary to evaluate the financial sustainability of these technologies in different contexts.

Training is essential for the success of telemonitoring. Healthcare professionals, patients, and their families must be trained in the use of these technologies, recognizing warning signs and knowing how to act in emergencies (25, 26). Research highlights the importance of comprehensive education to ensure effective use and adherence to these technologies. Postoperative patients often show higher adherence due to anxiety and fear of complications, a phenomenon well-documented in the literature (27).

The risks associated with using these technologies, especially for patients at high risk of severe complications, are significant. The implementation of alert and rapid response systems is essential to ensure immediate care when necessary (28, 29). Our practice included the creation of emergency protocols and integration with the institutional electronic health record for a rapid and effective response.

As these technologies evolve, data security becomes crucial to ensure patients feel safe and confident. Robust data protection measures were fundamental to maintaining trust and compliance with telemonitoring protocol (30, 31).

In addition to these issues, devices conventionally used for arrhythmia assessment face other challenges, such as frequent electrode replacements and user discomfort, especially due to the risk of disengagement, as demonstrated in the study by Zimetbaum & Goldman (32). The lack of real-time data analysis represents a crucial clinical limitation, compromising the ability for immediate intervention.

Therefore, the search for more comprehensive, practical, and less intrusive approaches is essential to ensure effective and safe healthcare for these patients.

In this scenario, smartwatches emerge as a promising solution (33). The use of this technology provides effective monitoring over long periods in an uncomplicated and fast manner, mitigating the risk of disengagement and providing a sense of security and control to patients (34).

It is known that the current global health monitoring landscape has undergone significant transitions as global technological advances continue. There is a growing emphasis on individuals playing a central role in managing their own health (35). However, for this approach to be even more effective, the integration of data from smartwatches into a digital platform is crucial for managing and organizing health data. Moreover, the possibility of connecting this platform to the electronic health record allows for long-term and detailed monitoring of a patient's clinical evolution over the years (36).

Thus, this investigation innovates by analyzing continuous and intermittent clinical monitoring data, being the first study to capture health parameters, with a focus on ECG readings, centered on the use of a smartwatch integrated into a digital telemonitoring platform. Our results demonstrated consistent credibility of the data provided by the device. Through agreement analysis, strong concordance was revealed between the cardiologist and the device-generated report for AF detection, and precision analysis showed a high sensitivity of 96.2% and an accuracy of 93.1%, providing valuable reports to support swift and early arrhythmia detection by the clinical team.

These findings are consistent with a previous proof-of-concept study of a similar device, which demonstrated a sensitivity of 98.0%, reinforcing that even for complex cardiac patients in a critical postoperative period, this approach maintained consistent measurement quality (37). These results have notable clinical contributions to the perspective of improving the surveillance and management of post-cardiac surgery AF in a prompt, reliable, and practical manner.

The clinical contributions of these results are notable, as they improve the surveillance and management of post-cardiac surgery AF in a prompt, reliable, and practical manner. Although our study did not reveal a reduction in ED visits, the precision and reliability of smartwatch data for AF detection underscore its potential to streamline postoperative care and improve patient outcomes. Future research could explore broader impacts of telemonitoring on healthcare resource utilization and patient quality of life, providing further insights into its benefits.

Studies like those by Zhu et al. (36) and Chen et al. (38) highlight the positive aspects of using smartwatches for health monitoring and engaging elderly patients, as well as the benefits of an integrated digital platform for medical care. These approaches represent a promising convergence of technology and medicine, with the potential to significantly improve clinical outcomes and the quality of life for cardiac patients.

Historical observations also support the potential of telemonitoring approaches to improve cardiovascular health, such as the pioneering 1996 study, which showed improved adherence and reduction in BP (39). In this context, a step forward was taken by a systematic review in 2013, which amalgamated findings from 23 clinical trials. that showed considerable advances in BP control, reducing the average S-BP by −4.71 mmHg and D-BP by −2.45 mmHg (40).

The results highlight the potential of telemonitoring to enhance cardiovascular health. As new health data acquisition methods gain relevance, concerns about parameter accuracy, including BP and other physiological measures, emerge.

Our study refutes these concerns, demonstrating that smartwatches are concordant and equivalent to standard methods for S-BP, D-BP, and HR. This aligns with criteria for BP measurement validation, surpassing the acceptable error limit (≤10 mmHg) and meeting stricter regulatory criteria with a maximum difference margin reference of ≤5 mmHg. Similarly, it is noted that HR measurements were concordant and achieved equivalence in our tests. On the other hand, SpO_2_ measurements were less reliable compared to traditional methods. Despite proven agreement in wearables' validation studies, our study faced significant challenges in collecting SpO_2_ data, with a high proportion of missing data (24%–36%, as observed in Supplementary Table S3. These missing data occurred due to consecutive failures in SpO_2_ readings for many patients in the study population. This finding, combined with the clinical peculiarity of our sample, which showed a notable incidence of AF, a factor not considered in the scientific validation studies of this parameter, suggests a hypothesis of compromised quality in assessing this parameter in the presence of arrhythmias or cardiac conditions.

Another important finding worth highlighting is the significant differences observed for the variables D-BP, HR, and SpO_2_ concerning the analysis time. These analyses indicated that there were changes in patients' health parameters regardless of the group or device used (smartwatch vs. standard). It's important to note that the patients were evaluated in Phase 4, one day after ICU discharge, which could have influenced the variability of vital signs. During this period, postoperative patients have various medications suspended, including those responsible for blood pressure and heart rate control. By Phase 6, patients had already been reintroduced to continuous-use medications adjusted as needed. Therefore, differences in measurements are expected between Phase 4 (after ICU discharge) and one month later (Phase 6) for both groups. It's noteworthy that with the help of available technological resources, we were able to conduct detailed monitoring of the patients in the TLM group. This capability was facilitated by continuous monitoring using smartwatches, as opposed to the conventional clinical follow-up represented by the CTL group. This highlights that the use of smartwatches allowed for meticulous tracking of changes in vital signs over the 30-day period. Furthermore, it's important to observe that, in our study, patient digital usability was not an issue. Despite the TLM group being more tech-savvy and younger, and the CTL group having a higher age and tech use frequency, there were no gender, skin color, or education level differences. It's worth noting that the TLM group, despite a significantly younger average age, included patients over 70 years old.

In the telemonitoring phase, challenges arose with a patient's severe tremors affecting ECG image stability. Techniques were employed to stabilize upper limbs, reducing tremor interference and improving ECG clarity. This adaptation highlighted smartwatches' versatility for conditions like Parkinson's disease, reinforcing their applicability in early disease identification through incorporated accelerometers (41).

A noteworthy discovery involved early intervention for asymptomatic bradyarrhythmia detected on the FAPO SI^3^ platform post-aortic valve replacement surgery, leading to pacemaker implantation. Approximately 6% of valve surgery patients may require a pacemaker, highlighting the importance of timely identification through smart devices.

While smartwatches play a central role in follow-up, they lack in-depth analysis. The FAPO-SI^3^ platform complements this by compiling wearable data, clinical records, and medical information for a comprehensive overview, facilitating precise medical decisions.

Utilizing Galaxy Watch5, the FAPO-SI^3^ platform provided real-time vital parameter acquisition with secure data storage through AWS and PostgreSQL. An intuitive dashboard aided healthcare professionals in understanding, identifying anomalies, supporting accurate assessments, and enabling continuous monitoring. Integration with monitoring devices and the SI^3^ electronic health record automated data collection, minimizing errors. Advanced analysis identified patterns, aiding healthcare decisions. The FAPO-SI^3^ platform efficiently acquired, stored, and visualized patient data, facilitating remote monitoring in the cardiac postoperative phase. Notable results include measurement accuracy, data security, diverse user inclusion, centralized information, and effective clinical monitoring, overcoming perceived barriers in advanced healthcare.

## 5 Conclusion

The seamless integration of smartwatches with a telemonitoring platform effectively monitored complex cardiac postoperative patients, providing reliable HR, BP, and AF detection. Despite no significant reduction in ED visits, telemonitoring enabled early clinical change detection and symptom management, potentially optimizing outcomes. Positive clinical results highlight its viability for advanced healthcare, suggesting future standardization in critical patient management. This study establishes a robust foundation for expanding telemonitoring in postoperative care. This study lays a robust foundation for the continued exploration and expansion of this promising medical care approach.

## Data Availability

The original contributions presented in the study are included in the article/Supplementary Material, further inquiries can be directed to the corresponding author.
