# Supplementary material for: Enhancing cardiac postoperative care: a smartwatch-integrated remote telemonitoring platform for health screening with ECG analysis
Source: Front Cardiovasc Med. 2024 Sep 23;11:1443998. doi: 10.3389/fcvm.2024.1443998 (PMC11460294; doi:10.3389/fcvm.2024.1443998)
Supplement: Supplementary file 1 [file Datasheet1.pdf]

SUPPLEMENTARY MATERIAL

Enhancing Cardiac Postoperative Care: A Smartwatch-Integrated Remote Telemonitoring Platform for Health Screening with ECG Analysis

1. Evaluation of the Impact of Telemonitoring with Smartwatches on Patient Perception

Supplementary Table 1. Technology Perception Questionnaire

Questionnaire	CTL n=53	TLM n=55	Total n=108
**Among the scenarios below, which one do you identify with the most:**			
I don't use technology	(1) 1.9%	(3) 5.6%	(4) 3.7%
Television and radio	(20) 37.7%	(7) 12.7%	(27) 25%
Computer and cellphone	(27) 50.9%	(33) 60%	(60) 55.6 %
I use multiple devices	(5) 9.4%	(12) 21.8%	(17) 15.7 %
**Assessing the perception of self-care after the study, how would you rate it?**			
It helped me learn more	(14) 26.4%	(7) 12.7%	(21) 19.4%
It gave me greater control	(17) 32.1%	(36) 65.5%	(53) 49.1%
It made my life easier	(13) 24.5%	(0) 0%	(13) 12%
All of the options	(1) 1.9%	(6) 10.9%	(7) 6.5%
Other option	(8) 15.1%	(5) 9.1%	(13) 12%
**Benefits of the FAPO study**			
Faster decision-making	(5) 9.4%	(2) 3.6 %	(7) 6.5 %
Better informed	(39) 73.6%	(19) 34.5%	(58) 53.7 %
Improved interaction with healthcare professionals	(4) 7.5%	(22) 40%	(26) 24.1 %
None	(4) 7.5 %	(1) 1.8%	(5) 4.6 %
All options	(1) 3.7%	(11) 20%	(12) 11.1 %
**How useful were the features of the FAPO-X watch and app?**			
Did not use	(53) 100%	(1) 1.9 %	(54) 50%
Not very useful	N/A	(0) 0.0 %	(0) 0%
Useful	N/A	(15) 27.3%	(15) 15.9%
Very useful	N/A	(39) 70.9%	(39) 36.1%
**How useful were the messages sent by the ChatBot?**			
Did not use	(53) 100%	(2) 3.6%	(55) 50.9%
Not useful at all	N/A	(1) 1.8%	(1) 0.9 %
Not very useful	N/A	(1) 1.8%	(1) 0.9%
Useful	N/A	(19) 34.5%	(19) 17.6%
Very useful	N/A	(32) 58.2%	(32) 26.6%
**Providing Health Readings**			
Did not use	(53) 100%	(1) 1.8%	(54) 50%
Not very useful	N/A	(2) 3.6%	(2) 1.9%
Useful	N/A	(16) 29.1%	(16) 14.8%
Very useful	N/A	(35) 63.6%	(35) 32.4%
I don't know	N/A	(1) 1.8%	(1) 0.9%
**Exercise guidance**			
Did not use	(53) 100%	(2) 3.8 %	(36) 34.3 %
Not useful at all	N/A	(4) 7.7 %	(22) 21.0 %
Not very useful	N/A	(5) 9.6 %	(5) 4.8 %
Useful	N/A	(19) 36.5 %	20(19.0 %)
Very useful	N/A	(22) 42.3 %	22(21.0 %)

I don't know	N/A	(0) 0%	(0) 0%
**Taking medications**			
Did not use	(53) 100%	(33) 60%	(86) 79.6%
Not useful at all	N/A	(9) 16.4%	(9) 8.3%
Not very useful	N/A	(2) 3.6%	(2) 1.9%
Useful	N/A	(7) 12.7%	(7) 6.5%
Very useful	N/A	(3) 5.5%	(3) 2.8%
I don't know	N/A	(1) 1.8%	(1) 0.9%
**Engaging in physical exercises**			
Did not use	(53) 100%	(2) 3.6%	(35) 33.3 %
Not useful at all	N/A	(5) 9.1%	(48) 45.7 %
Slightly useful	N/A	(5) 9.1%	(6) 5.7 %
Useful	N/A	(19) 34.5%	(10) 9.5 %
Very useful	N/A	(22) 40%	(6) 5.7 %
I don't know	N/A	(1) 1.8%	(1) 0.9
**How do you assess the importance of these smart watches for screening health data?**			
I don't know	(11) 20.8%	(0) 0%	(11) 20.4 %
Important	(20) 37.7%	(0) 0%	(21) 38.9 %
Very important	(20) 37.7%	(55) 100%	(22) 40.7 %
It doesn't matter	(2) 3.8%	(0) 0%	(2) 1.9%

Table S1. All values of the characteristics are expressed in number of patients (n) and percentage (%).
N/A: not applicable

2. Emergency Department Visits by Group

Contingency Table

	ED Visits		
**Group**	**No**	**Yes**	**Total**
CTL	35	12	47
TLM	36	12	48
Total	71	24	95

Chi-square tests (χ^2)

	Value	df	p
χ^2 with Continuity Correction	1.78e-31	1	1.000
N	95		

Supplementary Table 2. Emergency Department Visits by Group: Contingency table showing emergency department (ED) visits (No and Yes) for CTL (Control) and TLM (Telemonitored) groups, with χ^2 test results. (df): Degrees of freedom associated with the chi-square test. (χ^2 with Continuity Correction): Chi-square value adjusted for continuity correction to account for small sample sizes.

.

3. Bland Altman with Missing Data - Untreated Data

Supplementary Table 3. Bland-Altman Analysis Results from Telemonitored Group: Smartwatch vs. Gold Standard

Bland Altman – phase 4				
	**S-BP (mmHg)** n=49	**D-BP (mmHg)** n=49	**HR (bpm)** n=49	**SPO_2 (%)** n=33
Gold Standard (mean±SD)	116±13.8	76.6±9.31	89.8±12.2	93.7±3.03
Smartwatch (mean±SD)	116±13.7	75.3±9.55	90.6±12.5	91.2±3.58
Difference (mean±SD)	-0.03±3.03	-0.45±2.62	0.775±2.80	-2.51±4.68
Mean (Between Devices)	116	76.8	90.2	92.5
Lower limit of agreement	-5.9086	-4.678	-6.260	-7.08
Upper limit of agreement	5.9875	5.595	4.710	12.10
Single Sample T-test** (p value)	0.9	0.2	0.06	**0.006**
**Bland Altman – Phase 6**				
	**S-BP** n=51	**D-BP** n=51	**HR** n=51	**SPO_2** n=39
Gold Standard (mean±SD)	118±13.7	80.3±9.30	83.1±16.3	96.4±2.20
Smartwatch (mean±SD)	118±14.2	79.9±11.6	82.3±15.1	93.9±4.23
Difference (mean±SD)	-0.279±8.03	-0.389±6.87	-0.732±6.87	-2.54±4.36
Mean (Between Devices)	118	80.1	82.7	95.2
Lower limit of agreement	-15.467	-13.088	-12.595	-6.40
Upper limit of agreement	16.025	13.866	14.059	11.48
Single Sample T-test** (p value)	0.8	0.7	**0.4**	**0.001**

Table S3. S-BP refers to Systolic Blood Pressure, D-BP refers to Diastolic Blood Pressure, HR refers to Heart Rate, and SpO2 refers to Blood Oxygen Saturation (n= 55. per variable). SD represents Standard

Deviation. The values are presented in the format of Mean ± SD.

Supplementary Figure 1. Bland-Altman Analysis: Pre-Telemonitoring Phase - Smartwatch vs. Gold Standard

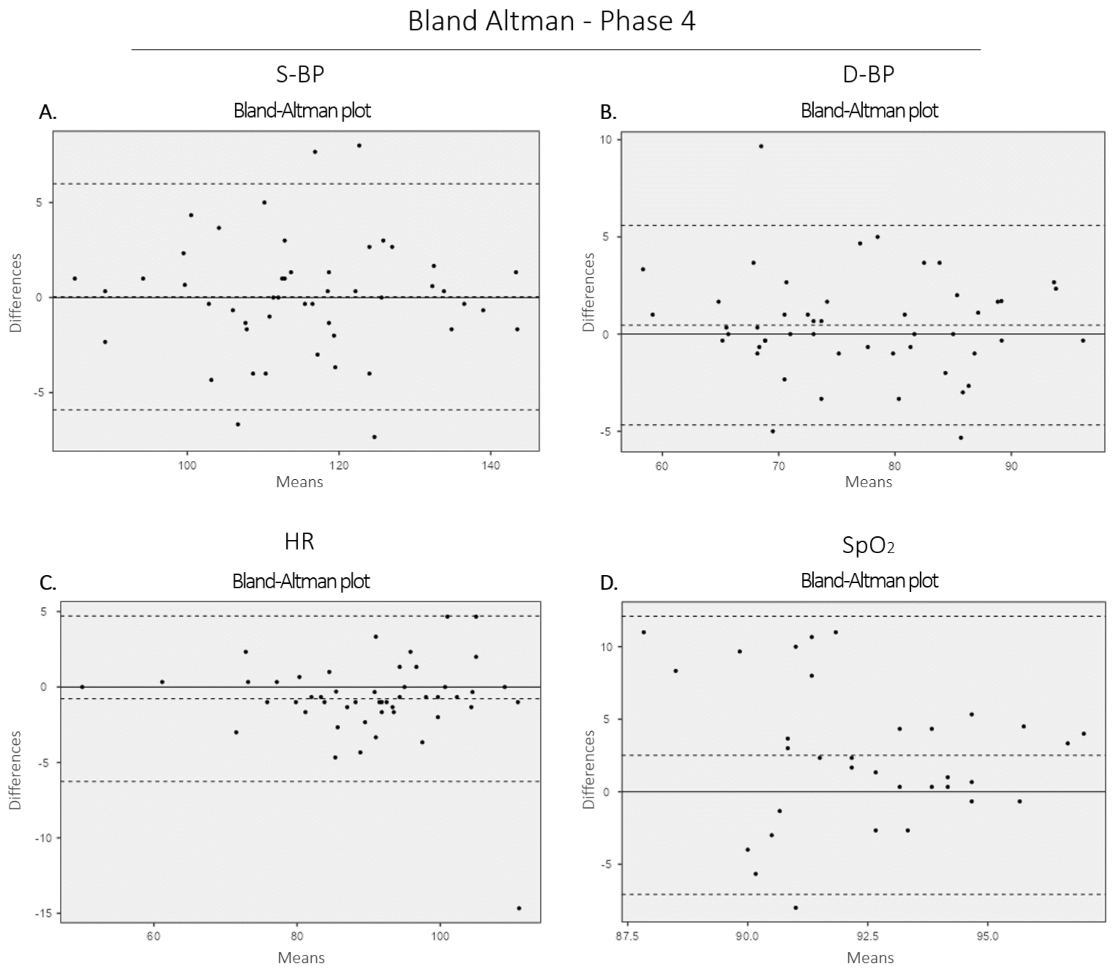

Figure S1. Bland-Altman Analysis Results for the Telemonitored Group (pre-telemonitoring phase): Smartwatch vs. Gold Standard Comparison. The plot showcases the agreement between measurements obtained from the smartwatch and the gold standard for various vital signs: (A) Systolic Blood Pressure, (B) Diastolic Blood Pressure, (C) Heart Rate, and (D) Oxygen Saturation.

Supplementary Figure 2. Bland-Altman Analysis: Post-Telemonitoring Phase - Smartwatch vs. Gold Standard

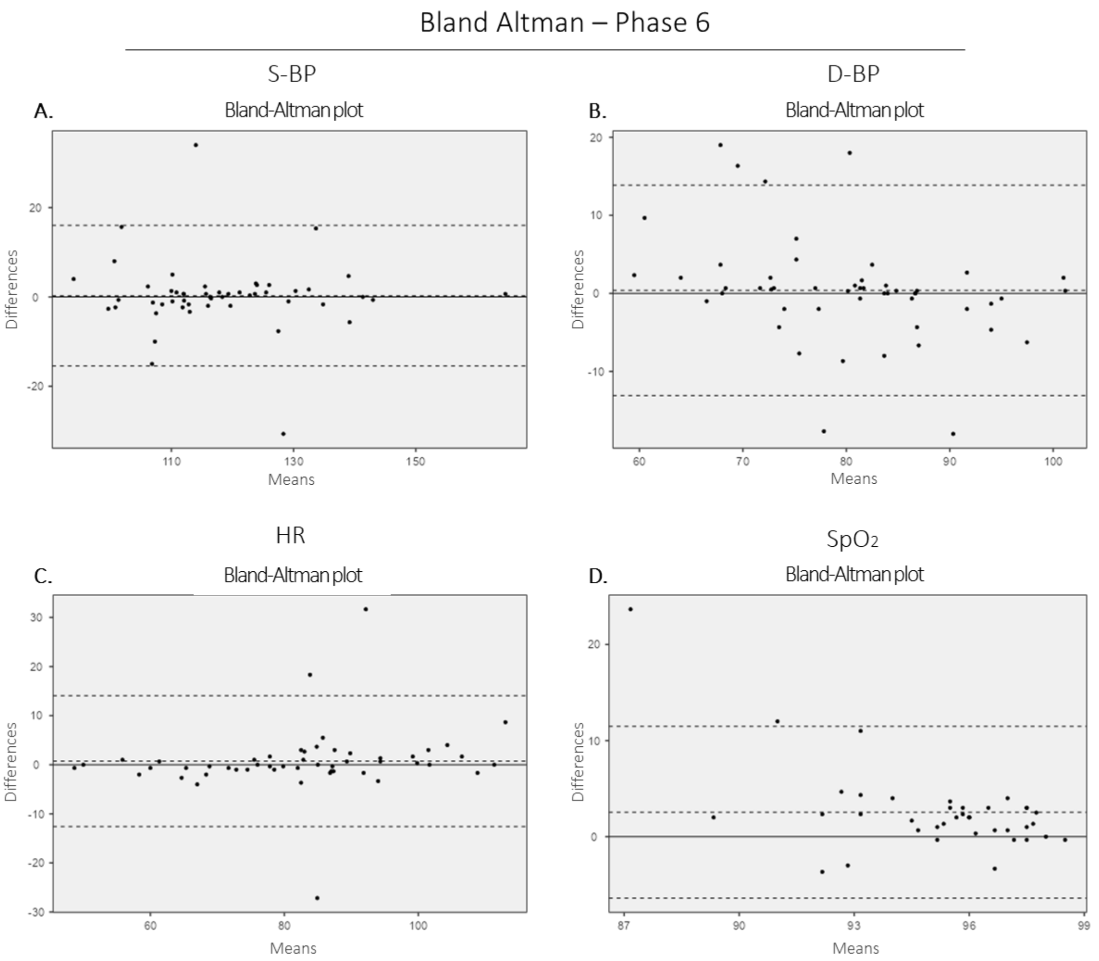

Figure S2. Bland-Altman Analysis Results for the Telemonitored Group (post-telemonitoring phase): Smartwatch vs. Gold Standard Comparison. The plot showcases the agreement between measurements obtained from the smartwatch and the gold standard for various vital signs: (A) Systolic Blood Pressure, (B) Diastolic Blood Pressure, (C) Heart Rate, and (D) Oxygen Saturation.

4. TOST test with Missing data - Untreated Data

Supplementary Table 4. TOST Results for Telemonitored in the Phase 4 - Vital Signs during Telemonitored Pre-Measurement Phase

TOST Results phase 4		t	df	p
**S-BP**	t-test	-0.00662	106	0.995
	TOST Upper	1.78	106	**0.039**
	TOST Lower	-1.79	106	**0.038**
**D-BP**	t-test	-0.226	106	0.822
	TOST Upper	2.5	106	**0.007**
	TOST Lower	-2.95	106	**0.002**
**HR**	t-test	0.358	106	0.721
	TOST Upper	2.96	106	**0.002**
	TOST Lower	-2.24	106	**0.014**
**SpO_2**	t-test	-3.37	70	**0.001**

Table S4. TOST Analysis for Pre-Measurement Phase: *Interpretation of TOST Upper and Lower only

Supplementary Figure 3. TOST Analysis for Pre-Measurement Phase

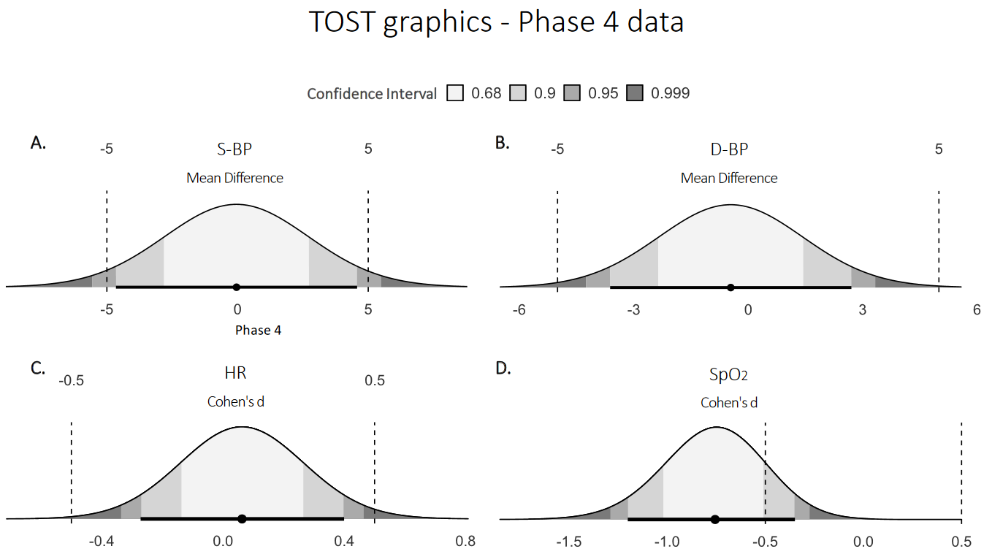

Figure S3. TOST Analysis for Pre-Measurement Phase. (A) Systolic Blood Pressure (S-BP), (B) Heart Rate (HR), (C) Diastolic Blood Pressure (D-BP) and (D) Oxygen Saturation (SpO2). Each subplot showcases the statistical analysis and equivalence testing for the respective vital sign.

Supplementary Table 5. TOST Results for Telemonitored Post-Measurement Phase

	TOST Results Phase 6	t	df	p
**S-BP**	t-test	-0.13	102	0.897
	TOST Upper	1.69	102	**0.047**
	TOST Lower	-1.95	102	**0.027**
**D-BP**	t-test	-0.0984	102	0.922
	TOST Upper	2.41	102	**0.009**
	TOST Lower	-2.61	102	**0.005**
**HR**	t-test	-0.203	102	0.839
	TOST Upper	2.35	102	**0.01**
	TOST Lower	-2.75	102	**0.003**
**SpO_2**	t-test	-3.23	80	**0.002**

Table S5. TOST Analysis for Post-Measurement Phase: *Interpretation of TOST Upper and Lower only

Supplementary Figure 4. TOST Analysis for Post-Measurement Phase

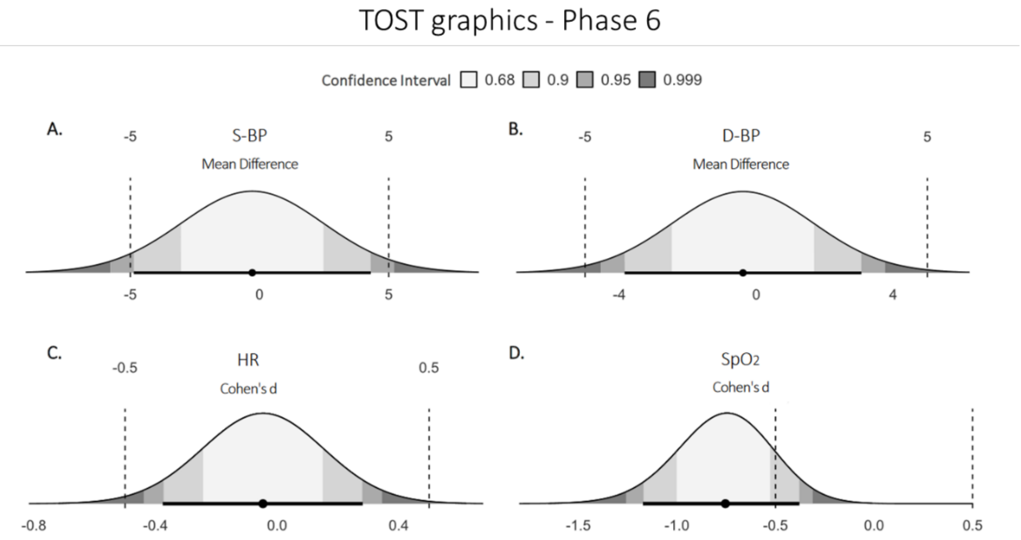

Figure S4. TOST Analysis for Post-Measurement Phase. (A) Systolic Blood Pressure (S-BP), (B) Heart Rate (HR), (C) Diastolic Blood Pressure (D-BP) and, (D) Oxygen Saturation (SpO_2). Each subplot showcases the statistical analysis and equivalence testing for the respective vital sign.
